# Prognostic nomogram integrated systemic inflammation score for patients with esophageal squamouscell carcinoma undergoing radical esophagectomy

**DOI:** 10.1038/srep18811

**Published:** 2015-12-22

**Authors:** Yingjie Shao, Zhonghua Ning, Jun Chen, Yiting Geng, Wendong Gu, Jin Huang, Honglei Pei, Yueping Shen, Jingting Jiang

**Affiliations:** 1 Department of Radiation Oncology, The Third Affiliated Hospital of Soochow University, 185 Juqian Street, Changzhou 213003, P.R. China; 2 Department of Oncology, The Third Affiliated Hospital of Soochow University, 185 Juqian Street, Changzhou 213003, P.R. China; 3 Jiangsu Key Laboratory of Preventive and Translational Medicine for Geriatric Diseases, School of Public Health, Soochow University, Suzhou 215123, China; 4 Department of Tumor Biological Treatment, The Third Affiliated Hospital of Soochow University, 185 Juqian Street, Changzhou 213003, P.R. China

## Abstract

Growing evidence indicates that nomogram combined with the biomarkers of systemic inflammation response could provide more accurate prediction than conventional staging systems in tumors. This study aimed to establish an effective prognostic nomogram for resectable thoracic esophageal squamouscell carcinoma (ESCC) based on the clinicopathological parameters and inflammation-based prognostic scores. We retrospectively investigated 916 ESCC patients who underwent radical esophagectomy. The predictive accuracy and discriminative ability of the nomogram were determined by concordance index (C-index) and calibration curve, and compared with the 6^th^ and 7^th^ AJCC TNM classifications. The neutrophil lymphocyte ratio (NLR), C-reactive protein albumin (CRP/Alb) ratio, histological grade, T stage and modified N stage were integrated in the nomogram. The C-index of the nomogram for predicting the survival was 0.72, which showed better predictive ability of OS than the 6^th^ or 7^th^ TNM stages in the primary cohort (*P* < 0.001). The calibration curve showed high consistency between the nomogram and actual observation. The decision curve analysis showed more potential of clinical application of the prediction models compared with TNM staging system. Moreover, our findings were supported by the validation cohort. The proposed nomogram showed more accurate prognostic prediction for patients with ESCC after radical esophagectomy.

Esophageal cancer is a common cause of cancer death worldwide^1^, it is also the 5th leading cancer in incidence and 4th in mortality in China^2^. In 2010, there were 287,632 new cases and 208,473 deaths of esophageal cancer in China^2^. In despite of the development of comprehensive treatment strategies, 5-year overall survival (OS) rate for esophageal cancer is 15–35% and the prognosis remains dismal^3^,^4^. Esophageal cancer is divided into two main pathological subtypes: squamous cell carcinoma and adenocarcinoma. The predominant histological type of esophageal cancer in China is esophageal squamous cell carcinoma (ESCC), which accounts for over 90% of cases^5^. Traditionally, the prognosis of ESCC was performed according to the 6^th^ and 7^th^ edition American Joint Committee on Cancer tumor-node-metastasis (AJCC TNM) staging system^6^. However, ESCC patients at the same TNM stage and received similar therapy usually had variable outcomes^7^, suggesting that the current AJCC staging system that only assesses anatomical factors may be inadequate to make a treatment decision and evaluate the prognosis. Therefore, there is an urgent demand for a new tool that can provide reliable prognostic information in individual patient.

The visual format of nomogram is a simple and advanced prediction model that estimates the survival of individual patient by incorporating multiple clinical variables and their interdependent relationships^8^. The nomogram has been extensively used for many cancers, and it has been proposed as an alternative or even as a new standard^9^,^10^,^11^,^12^,^13^,^14^. Recently, several studies reported that nomogram combined with the biomarkers of systemic inflammation response could provide more accurate prediction than conventional staging systems in a variety of tumors^15^,^16^,^17^,^18^. The systemic inflammation-based prognostic scores, including the Glasgow Prognostic Score (GPS), modified GPS (mGPS), C-reactive protein albumin (CRP/Alb) ratio, neutrophil lymphocyte ratio (NLR), platelet lymphocyte ratio (PLR) and lymphocyte monocyte ratio (LMR) have emerged as prognostic factors in ESCC^19^,^20^. Compared with other numerous prognostic factors, the inflammation-based prognostic scores are simple, inexpensive and widely available from preoperative evaluation of blood test. However, there is few study establishing a prognostic nomogram for ESCC based on these biomarkers. This study aimed to establish a prognostic nomogram for resectable thoracic ESCC based on the clinicopathological parameters and the inflammation-based prognostic scores, to determine whether this model provides more accurate prediction of patient survival compared with the 6^th^ and 7^th^ edition of AJCC TNM classifications.

## Results

### Clinicopathological characteristics of patients

The clinicopathological characteristics of patients in the primary cohort (n = 633) and validation cohort (n = 283) are listed in Table 1. The ratio of men to women in the primary and validation cohort was 3.26:1 and 2.94:1, respectively. The median age in the primary and validation cohorts was 60 years (range, 37–83 years) and 61 years (range, 38–84 years), respectively. In the primary cohort, the median OS was 40 months (range, 3 to 146.2 months) and the rate of 3- and 5-year OS was 53.1% and 43.2%, respectively. In the validation cohort, the median OS was 44 months (range, 3 to 82 months), and the rate of 3- and 5-year OS was 54.4% and 44.6%, respectively.

### Nomogram development and internal validation

**Figure 1:**
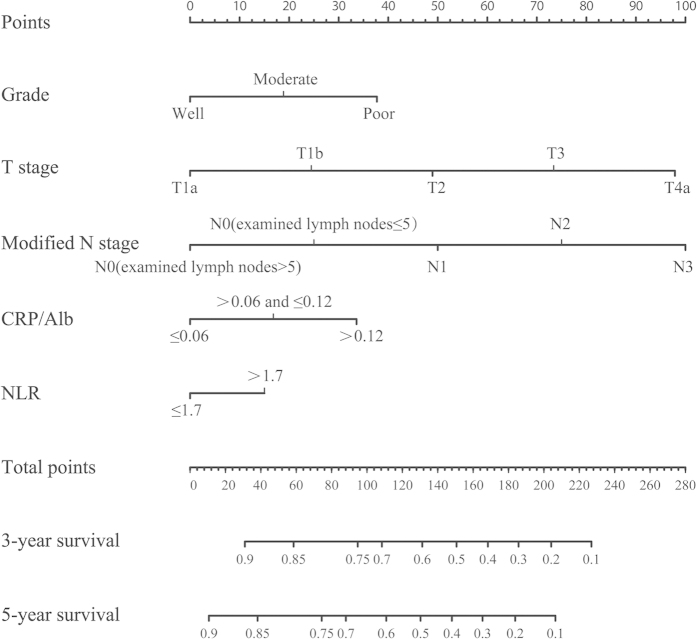
Evaluation of nomogram integrated systemic inflammation scores in the patients with esophageal squamous cell cancer after radical esophagectomy. To use the nomogram, the value attributed to an individual patient is located on each variable axis, and a line is drawn upwards to determine the number of points received for each variable value. The sum of these numbers is located on the total points axis, and a line is drawn downward to the survival axis to determine the likelihood of 3- or 5-year survival. CRP/Alb, C-reactive protein/albumin; NLR: neutrophil lymphocyte ratio.

**Figure 2:**
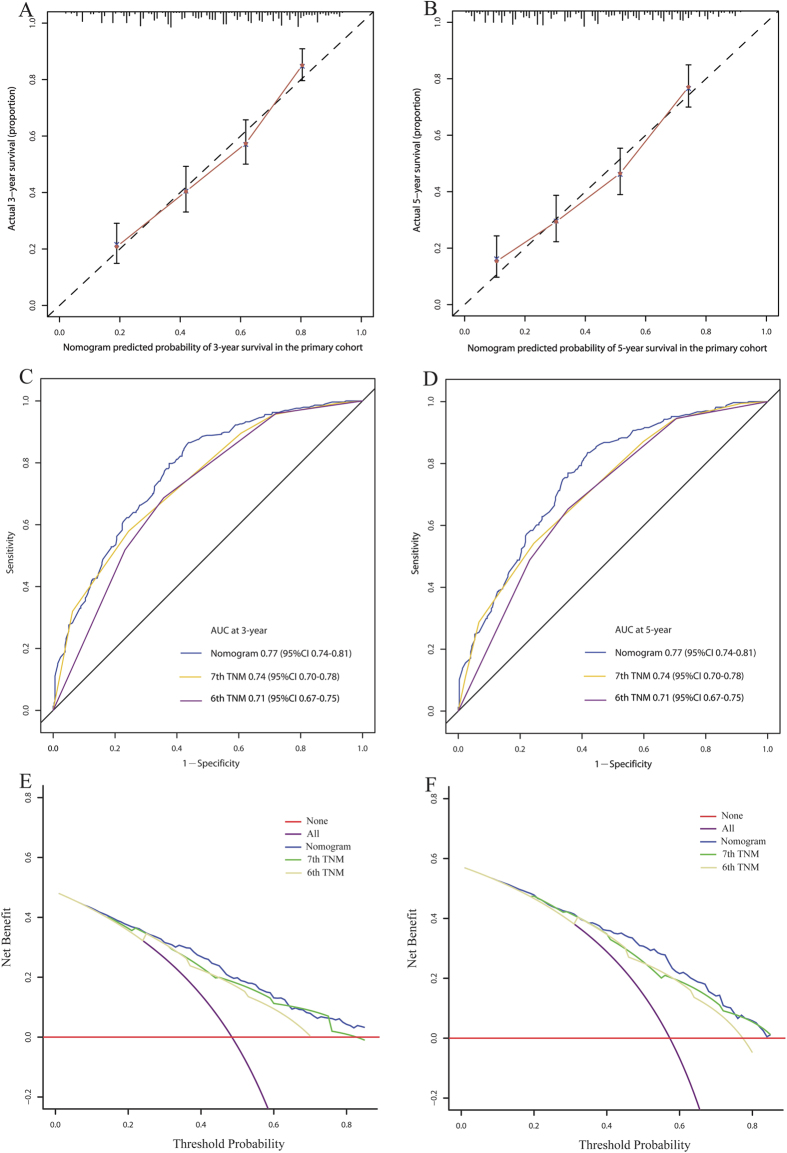
The calibration curve for predicting patient survival at 3-year (A) and 5-year (B) in the primary cohort. Time-dependent receiver operating characteristic (ROC) curves by nomogram, 6^th^ AJCC-TNM staging system and 7^th^ AJCC-TNM staging system for 3-year (**C**) and 5-year (**D**) OS in the primary cohort. Decision curve analyses by nomogram, 6^th^ AJCC-TNM staging system, and 7^th^ AJCC-TNM staging system for 3-year (**E**) and 5-year (**F**) OS in the primary cohort.

In univariate analysis, histological grade, T stage, modified N stage, PLR, NLR, LMR, GPS, mGPS and CRP/Alb were found to be significant prognostic factors, while age, sex, body mass index (BMI), tumor location, tumor length and examined lymph nodes showed no statistical differences (Table 2). We explored the association among these inflammation-based prognostic scores. It was found that the classifications of GPS, mGPS and CRP/Alb were highly correlated. Thus three separate multivariate models (GPS, mGPS and CRP/Alb) were run to avoid problems with the presence of multicollinearity. Multivariate analyses demonstrated that histological grade, T stage, modified N stage, NLR, GPS, mGPS and CRP/Alb were independent risk factors for OS (Table 2). Backward stepwise selection with the Akaike information criterion (AIC) in Cox proportional hazards regression modeling was used to find a best-fit model among these independent risk factors. Finally, the nomogram that integrated five variables: histological grade, T stage, modified N stage, NLR and CRP/Alb was used to predict 3- and 5-year OS in the primary cohort (Fig. 1). The concordance index (C-index) for OS prediction was 0.72 (95% CI, 0.69–0.75). The calibration plot for the probability of survival at 3 or 5 years after surgery showed a good correlation between the prediction by nomogram and actual observation (Fig. 2A,B).

### Comparison of predictive accuracy for OS between nomogram and conventional staging systems

**Figure 3:**
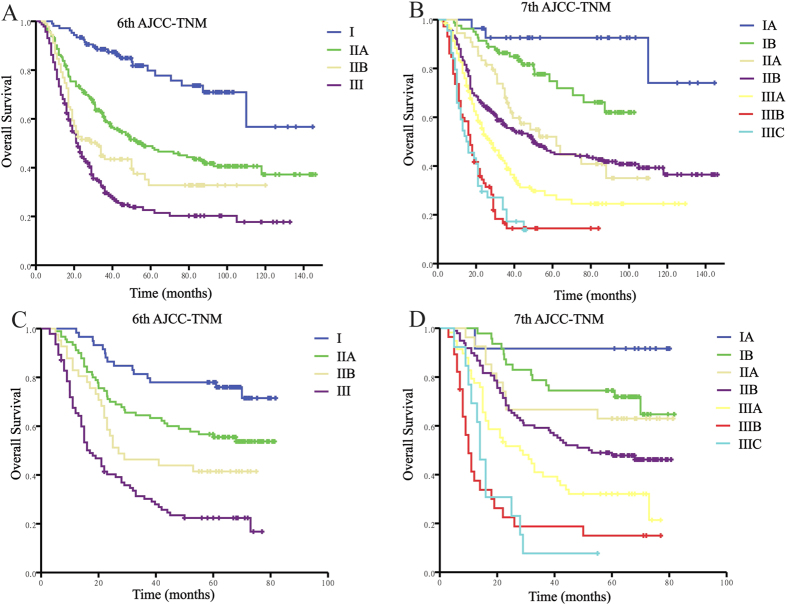
Kaplan-Meier curve of the primary cohort stratified for 6^th^ AJCC-TNM staging system (A) and 7^th^ AJCC-TNM staging system (B). Kaplan-Meier curve of the validation cohort stratified for 6^th^ AJCC-TNM staging system (**C**) and 7^th^ AJCC-TNM staging system (**D**).

As shown in Fig. 3, the 6^th^ and 7^th^ AJCC classifications showed good prognostic stratification for most patients. However, the 7^th^ AJCC classifications were unsatisfactory in stratifying patients between stages IIIB and IIIC, while the 6^th^ AJCC classifications were unsatisfactory in stratifying patients between stages IIA and IIB.

Our nomogram displayed better accuracy for predicting the survival in the primary cohort. The C-index of the nomogram was 0.72, which was significantly higher than that of the 7^th^ AJCC staging system (0.68) and the 6^th^ AJCC staging system (0.66) (*P* < 0.001). The time-dependent receiver operating characteristics (ROC) curve showed higher sensitivity and specificity for predicting OS at 3- and 5-year of follow-up (Fig. 2C,D). In the decision curve analysis, the nomogram demonstrated high potential of clinical application because it ensured better net benefits throughout the entire range of threshold probabilities for survival after 3 or 5 years compared with the TNM staging systems (Fig. 2E,F). These results suggest that our nomogram has better performance for predicting OS than the AJCC TNM classifications.

### Validation of predictive accuracy of the nomogram for OS

**Figure 4:**
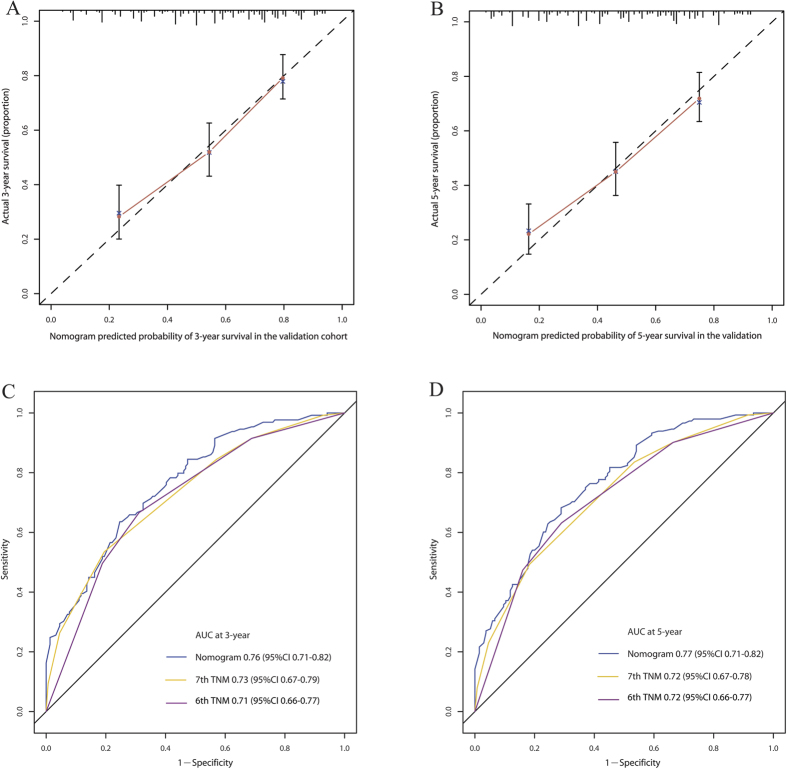
The calibration curve for predicting patient survival at 3-year (A) and 5-year (B) in the validation cohort. Time-dependent receiver operating characteristic (ROC) curves by nomogram, 6^th^ AJCC-TNM staging system and 7^th^ AJCC-TNM staging system for 3-year (**C**) and 5-year (**D**) OS in the validation cohort.

Calculation of OS was done using the designed nomogram on each patient in the validation cohort. The calibration curves showed good consistency in the probability of 3- and 5-year survival between the actual observation and the nomogram prediction (Fig. 4A,B). The C-index of the nomogram for predicting OS was 0.71 (95% CI, 0.67 to 0.77) in the validation cohort, which was also significantly higher than the C-index (0.68) of the 6^th^ TNM classification (*P* < 0.001) and the C-index (0.69) of the 7^th^ TNM classification (*P* < 0.001). The ROC curve also showed the similar results (Fig. 4C,D). These results suggest that the nomogram is a more accurate and useful tool for the prediction of OS in patients with resectable ESCC.

## Discussion

In recent years, nomograms have been constructed in many malignancies, and some of these nomograms have been found to be more reliable prediction than the traditional staging system^9^,^10^,^11^,^12^,^13^,^14^. Despite many advantages, there is few study on the prognostic nomogram design for resectable ESCC patients. However, that nomogram did not match well to our patient cohort. In this study, the clinicopathological variables including histological grade, T stage, modified N stage, NLR and CRP/Alb ratio were integrated in a prognostic nomogram. This nomogram predicted OS with an accuracy of C-index 0.72, which showed significantly better prediction of OS than the 6^th^ or 7^th^ TNM staging system in the primary cohort. The ROC curve also showed higher sensitivity and specificity for predicting 3- and 5-year OS compared with the 6^th^ or 7^th^ TNM staging system. These results were subsequently validated by an independent external data set. The calibration plots from both the primary and validation cohorts revealed good correlation between the predicted survival probability and the actual survival rate. The decision curve analysis showed more potential of clinical application of the prediction models compared with TNM staging system. Moreover, the information of CRP/Alb ratio and NLR can be obtained from the peripheral blood tests that were routinely conducted during preoperative examinations. Therefore our nomogram is a reliable tool to predict survival in resectable ESCC and is helpful to make individualized treatment decision.

Compared with Su’s nomogram^21^, our prediction model only included three clinicopathological factors: histological grade, T stage and modified N stage. Other clinicopathological factors such as tumor length and number of examined lymph nodes are not independent risk factors due to low associated hazard ratio (almost close to 1) in our study. It is worth mentioning that modified N stage instead of N stage was integrated in our nomogram. Our study found the count of examined lymph nodes was associated with the survival of the node-negative ESCC patients. Patients’ survival is positively correlated with the increasing number of negative lymph nodes for cancer examination. It is well accepted that small number of resected lymph nodes may miss positive lymph nodes and lead to the incorrect diagnosis^22^,^23^, but excessive lymphadenectomy will increase the risk of complications, such as anastomotic leakage, recurrent laryngeal nerve damage and respiratory complications^24^. In addition, extensive lymphadenectomy would lead to poor immune function and slow the postoperative recovery^25^. So the lymph node-negative ESCC patients (N0 stage patients) were divided into two groups based on examined 5 lymph nodes in our study.

Our nomogram also included the inflammation-based prognostic scores. Although the inflammation-based prognostic scores are not included in traditional staging systems, their roles in increasing predictive performance have been observed recently. The relationship between inflammation and tumor was first reported in 1863^26^. Over the past decades, accumulating evidence has indicated that inflammation contributes to tumor growth, progression and metastasis^27^. Recently, the systemic inflammatory response biomarkers such as acute-phase proteins and circulating immune cells have been found to be independent markers of prognosis in a variety of cancers^28^,^29^,^30^,^31^, including ESCC^19^,^20^. Albumin and CRP are accepted markers of acute-phase proteins. The most common prognostic scores based on serum CRP and albumin concentrations are GPS and mGPS. Besides GPS and mGPS, CRP/Alb ratio can be used as an independent prognostic factor in cancer^20^,^32^. The GPS, mGPS and CRP/Alb were all independent prognostic biomarkers with high correlation in our study. However, when classified by the mGPS and GPS in our primary cohort, 86.4% and 94.3% of patients were classified in the group of score 0. In the validation cohort, more than 90% patients with a score of 0 were classified by the mGPS and GPS. Therefore, GPS and mGPS apply only to a small group of patients, and have little clinical significance. Furthermore, backward stepwise selection chose the CRP/Alb ratio instead of GPS and mGPS to build the best-fit prediction model. So the CRP/Alb is superior to GPS and mGPS in our study. The PLR, NLR and LMR are the common prognostic scores based on circulating immune cells. In our study, the NLR, PLR and LMR were significant prognostic factors in univariate analysis, in while only the NLR was an independent prognostic factor for OS in multivariate analysis. Finally, the CRP/Alb ratio and NLR were included in our nomogram. Recently, Liu *et al.* built a nomogram based on various inflammatory biomarkers for respectable ESCC^33^. However, the nomogram of their study contained PLR, LMR and GPS, but not CRP/Alb radio and NLR. We found better prognostic effect of CRP/Alb radio than GPS for respectable ESCC, which was also confirmed by many recent articles^20^,^32^,^34^,^35^. For this reason, we use CRP/Alb radio instead of GPS. In addition, Liu *et al.* did not put the NLR included into the nomogram, because the multivariate analysis indicated the NLR not an independent prognostic factor. But significant prognostic effect was observed in the univariate analysis. This may be caused by the deficiency of correlation analysis among NLR, PLR and LMR before multivariate analysis. Moreover, Liu *et al.* constructed a nomogram without the necessary process of Performance and Application^8^,^36^. In this study we conducted such a supplement to make the results more scientific and reliable.

Several potential mechanisms can probably be used to explain the prognostic values of the inflammatory biomarkers in cancer: Firstly, C-reactive protein (CRP) and neutrophile granulocytes were triggered by cancer-related inflammatory factors, such as interleukin-6 (IL-6), tumor necrosis factor (TNF) and myeloid growth factors^37^,^38^. These inflammatory mediators facilitate the growth, invasion, metastasis and angiogenesis of tumor, disrupt host immune response, and induce the resistance to cytotoxic drugs^26^,^39^,^40^. Secondly, elevated neutrophils can secrete plenty of nitric oxide, arginase, and reactive oxygen species (ROS), leading to T cell activation disorders^41^. Meanwhile, increased circulating neutrophils have been reported to produce vascular endothelial growth factor (VEGF), causing tumor angiogenesis^42^,^43^. Thirdly, circulating monocytes determine the number of macrophages in tumor tissue, and the density of tumor-associated macrophages has been proven to correlate with angiogenesis, tumor invasion and poor prognosis^39^,^44^.

Although our nomogram demonstrated good predictive accuracy for survival, there are still several limitations in this study. First, the nomogram was established based on the data from an individual institution in China. Second, our study was a retrospective study, and there may exist selection bias during retrospective data collection. Third, there was heterogeneity in the reported thresholds that were used to define an elevated the inflammation-based prognostic scores in the literature. Therefore, our results need to be further verified in a prospective, large-scale collaborative study.

In conclusion, our proposed nomogram integrated the systemic inflammation scores can accurately predict the prognosis of patients with ESCC after radical esophagectomy. We believe that our nomograms would facilitate making the therapeutic decision and individualized patient counseling.

## Materials and Methods

### Patients

The study included 916 resectable ESCC patients who underwent radical esophagectomy in the Third Affiliated Hospital of Soochow University (Changzhou, China) from January 2002 to December 2012. Transthoracic esophagectomy with mediastinal and abdominal two-field lymphadenectomies was carried out in the present study. The inclusion criteria are as follows: radical thoracic ESCC, R0 resection, no combined malignancy, no distant metastasis, no preoperative or postoperative radiotherapy and/or chemotherapy. Among all enrolled patients, 283 patients from January 2008 to December 2009 were enrolled in the external validation cohort of this study, while the other patients were included in the primary cohort. The study protocol was performed in accordance with the guidelines outlined in the Declaration of Helsinki and was approved by the Ethics Committee of Third Affiliated Hospital of Soochow University. Written informed consent was obtained from all participants.

According to clinical findings or statistical methods, clinical data were collected and categorized as follows: age (≤60, >60), sex (male, female), BMI (<18.5, 18.5–24.5, >24.5), tumor location (upper, middle, and lower), histological grade (well, moderately, and poorly or not differentiated), tumor length (≤4 cm, 4–8 cm, >8 cm), T stage according to 7th edition of AJCC TNM staging (T1a-lamina propria or muscularis mucosae, T1b-submucosa, T2-superficial and deep muscular layer, T3-adventitia, T4a-pleura, pericardium, diaphragm, or adjacent, T4b-other unresectable adjacent structures), number of examined lymph nodes (≤5, 6–15, >15). To maximize the performance of the nomogram, we chose modified N stage instead of N stage. The modified N stage was defined as N stage in the 7th AJCC stage system except N0 stage. According to examined lymph nodes, the N0 stage was divided into two categories in the modified N stage. Thus the modified N stage was divided into N0 (examined lymph nodes > 5), N0 (examined lymph nodes ≤ 5), N1 (positive lymph nodes 1-2), N2 (positive lymph nodes 3–6) and N3 (positive lymph nodes ≥ 7).

All peripheral blood was collected and tested for neutrophils, lymphocytes, platelet, monocyte counts, serum C-reactive (CRP) and albumin levels just before operation. The inflammation-based prognostic scores in this study were defined and calculated as follows: (1) GPS, patients with both CRP >10 mg/L and albumin <35 g/L were allocated a score of 2; patients with CRP > 10 mg/L or albumin <35 g/L were allocated a score of 1; and patients with both CRP <10 mg/L and albumin>35 g/L were allocated a score of 0. (2) mGPS, patients with CRP < 10 mg/L were allocated a score of 0; patients with CRP > 10 mg/L or albumin >35 g/L were allocated a score of 1; patients with both CRP > 10 mg/Land albumin< 35 g/L were allocated a score of 2. Optimal cutoff values including NLR (NLR≤1.7, NLR>1.7), PLR (PLR ≤ 120, PLR > 120), LMR (LMR ≤ 3.57, LMR > 3.57) and CRP/Alb (CRP/Alb ≤ 0.06, 0.06 < CRP/Alb ≤ 0.12, CRP/Alb > 0.12) were determined by using X-tile software (http://www.tissuearray.org/rimmlab)^45^.

### Follow-Up

Follow-up was conducted as previously described^46^. All patients were followed up every three months in the first 2 years, every six months until 5 years, and then once annually. All patients underwent clinical, laboratory, imaging, and endoscopy examinations for assessing recurrence or metastasis.

The latest follow-up was conducted at the end of December 2014. All patients were followed up by phone calls and regular letters. The observation time in this study was the interval from the date of surgical resection to death or latest follow-up. Survived patients were censored on the day of the last follow-up. OS was determined by the period from the time of surgery to the last follow- up or date of patient death. The median follow-up was 39 months (range, 3 to 146.2 months).

### Statistical Analysis

Statistical analysis was carried out using SPSS 17.0 for windows (SPSS, Chicago, IL) and R software version 3.2.0 (http://www.r-project.org/) with Hmisc, rms, and survival ROC packages. Survival curves were made using the Kaplan-Meier method and compared using the log-rank test. All variables that achieved significance at *P* < 0.05 in univariate analyses were enrolled in multivariate Cox’s proportional hazards model. The nomogram was formulated based on the results of multivariate analysis. A final model selection was performed using a backward stepdown selection process with the AIC^47^. To evaluate the nomogram performance, we assessed both the discrimination and calibration of these models. The analysis of time-dependent ROC curve and C-index were used to compare the discrimination power for OS between different models. Confidence intervals (CIs) were obtained by creating 1000 bootstrap samples from the entire dataset and replicating the estimation process. The larger the C-index, the more accurate was the prognostic prediction^48^. Decision curve analysis was used to evaluate the clinical application of prediction models by quantifying the net benefits^49^. During the external validation of the nomogram, the total points of each patient in the validation cohort were calculated according to the generated nomogram, then Cox regression in this cohort was performed using the total points as a factor, and the C-index and calibration curve were finally derived based on the regression analysis. Nomogram construction and validation were performed with nomogram guide^8^,^36^. A *P* value less than 0.05 was considered to be statistically significant unless otherwise specified.

## Additional Information

**How to cite this article**: Shao, Y. *et al.* Prognostic nomogram integrated systemic inflammation score for patients with esophageal squamouscell carcinoma undergoing radical esophagectomy. *Sci. Rep.* **5**, 18811; doi: 10.1038/srep18811 (2015).

## Figures and Tables

**Table 1:** Clinicopathological characteristics and inflammation-based prognostic scores of patients with esophageal squamous cell carcinoma.

Characteristic	Primary Cohort (n = 633)		Validation Cohort (n = 283)	
	No. of Patients	%	No. of Patients	%
Sex				
Male	484	76.5	211	74.6
Female	149	23.5	72	25.4
Age				
≤60	317	50.1	138	48.8
>60	316	40.9	145	51.2
BMI				
<18.5	76	12.0	31	11.0
18.5–24.5	492	77.7	211	74.6
>24.5	65	10.3	41	14.4
Tumor location				
Upper	33	5.2	24	8.5
Middle	428	67.6	171	60.4
Lower	172	27.2	88	31.1
Histological grade				
Well differentiated	31	4.9	14	4.9
Moderately differentiated	325	51.3	126	44.5
Poorly or not differentiated	277	43.8	143	50.5
Tumor length (cm)				
<4	279	44.1	114	40.3
4–8	259	40.9	113	39.9
>8	95	15.0	56	19.8
T stage				
T1a	34	5.4	21	7.4
T1b	103	16.3	50	17.7
T2	166	26.2	59	20.8
T3	313	49.4	151	53.4
T4a	17	2.7	2	0.7
Examined lymph nodes				
≤5	163	25.7	36	12.7
6–15	343	54.2	171	60.4
>15	127	20.1	76	26.9
Modified N stage				
N0 (examined lymph nodes >5)	196	31.0	123	43.5
N0 (examined lymph nodes ≤5)	123	19.4	30	10.6
N1 (Positive lymph nodes 1–2)	187	29.5	83	29.3
N2 (Positive lymph nodes 3–6)	97	15.3	35	12.4
N3 (Positive lymph nodes ≥7)	30	4.7	12	4.2
TNM stage (AJCC, 6^th^)				
I	106	16.7	59	20.8
IIa	203	32.1	90	31.8
IIb	92	14.5	41	14.5
III	232	36.7	93	32.9
TNM stage (AJCC, 7^th^)				
Ia	28	4.4	12	4.2
Ib	81	12.8	47	16.6
IIa	54	8.5	27	9.5
IIb	216	34.1	98	34.6
IIIa	138	21.8	58	20.5
IIIb	72	11.4	28	9.9
IIIc	44	7.0	13	4.6
PLR				
≤120	338	53.4	120	42.4
>120	295	46.6	163	57.6
NLR				
≤1.7	192	30.3	98	34.6
>1.7	441	69.7	185	65.4
LMR				
≤3.57	283	44.7	137	48.4
>3.57	350	55.3	146	51.6
CRP/Alb				
≤0.06	206	32.5	170	60.1
>0.06 and ≤0.12	287	45.3	90	31.8
>0.12	140	22.1	23	8.1
GPS				
0	547	86.4	263	92.9
1	76	12.0	18	6.4
2	10	1.6	2	0.7
mGPS				
0	597	94.3	274	96.8
1	26	4.1	72	2.5
2	10	1.6	2	0.7

BMI: body mass index; PLR: platelet lymphocyte ratio; NLR: neutrophil lymphocyte ratio; LMR: lymphocyte monocyte ratio; CRP/Alb - C-reactive protein/albumin; GPS: glasgow prognostic score, mGPS – modified GPS; AJCC: American Joint Committee on Cancer

**Table 2:** Univariate and multivariate cox regression analyses for overall survival in patients with esophageal squamous cell carcinoma.

Variables	Univariate analysis		Multivariate analysis	
	HR (95% CI)	*P* value	HR (95% CI)	*P* value
Sex				
Male vs. Female	1.14 (0.88–1.47)	0.313		
Age				
≤60 years vs. >60 years	0.88 (0.72–1.09)	0.25		
BMI		0.246		
<18.5	Ref.			
18.5–24.5	0.75 (0.49–1.14)	0.174		
>24.5	0.77 (0.55–1.06)	0.103		
Histological grade		<0.001		0.003
Well differentiated	Ref.	—	Ref.	
Moderately differentiated	2.60 (1.41–4.79)	0.002	1.81 (0.97–3.35)	0.061
Poorly or not differentiated	3.87 (2.10–7.12)	<0.001	2.37 (1.27–4.43)	0.007
Tumor location		0.116		
Upper	Ref.			
Middle	1.65 (0.96–2.82)	0.070		
Lower	1.42 (0.81–2.49)	0.225		
Tumor length (cm)		0.279		
≤4	Ref.			
4–8	1.06 (0.85–1.34)	0.539		
>8	1.28 (0.95–1.73)	0.110		
T stage		<0.001		<0.001
T1	Ref.		Ref.	
T2	1.62 (1.12–2.35)	0.011	1.42 (0.97–2.08)	0.075
T3	3.31 (2.39–4.58)	<0.001	2.08 (1.48–2.94)	<0.001
T4	4.40 (2.37–8.19)	<0.001	3.38 (1.79–6.40)	<0.001
Examined lymph nodes		0.199		
≤5	Ref.			
6–15	1.00 (0.78–1.28)	0.992		
>15	1.27 (0.93–1.71)	0.129		
Modified N stage		<0.001		<0.001
N0 (examined lymph nodes >5)	Ref.		Ref.	
N0 (examined lymph nodes ≤5)	1.89 (1.35–2.64)	<0.001	1.93 (1.37–2.72)	<0.001
N1 (positive lymph nodes 1–2)	2.06 (1.60–2.65)	<0.001	2.06 (1.50–2.83)	<0.001
N2 (positive lymph nodes 3–6)	3.67 (2.75–4.89)	<0.001	3.40 (2.38–4.87)	<0.001
N3 (positive lymph nodes ≥7)	5.52 (3.63–8.41)	<0.001	5.66 (3.52–9.10)	<0.001
PLR				
>120 vs. ≤120	1.31 (1.06–161)	0.013	1.14 (0.92–1.42)	0.238
NLR				
>1.7 vs. ≤1.7	1.42 (1.11–1.80)	0.005	1.26 (1.01–1.64)	0.049
LMR				
≤3.57 vs. >3.57	1.30 (1.05–1.60)	0.015	1.11 (0.88–1.40)	0.373
CRP/Alb		<0.001		0.002
≤0.06	Ref.		Ref.	
>0.06 and ≤0.12	1.29 (1.00–1.66)	0.047	1.32 (1.02–1.70)	0.033
>0.12	1.92 (1.45–2.54)	<0.001	1.67 (1.26–2.22)	<0.001
GPS		<0.001		0.001
0	Ref.		Ref.	
1	1.53 (1.14–2.07)	0.005	1.38 (1.02–1.88)	0.037
2	3.88 (1.92–7.85)	<0.001	3.53 (1.70–7.31)	0.001
mGPS		<0.001		0.003
0	Ref.		Ref.	
1	1.45 (0.90–2.33)	0.127	1.24 (0.77–2.01)	0.373
2	3.74 (1.85–7.55)	<0.001	3.37 (1.63–6.97)	0.001

BMI: body mass index; PLR: platelet lymphocyte ratio; NLR: neutrophil lymphocyte ratio; LMR: lymphocyte monocyte ratio; CRP/Alb: C-reactive protein/albumin; GPS: glasgow prognostic score; mGPS: modified GPS, AJCC: American Joint Committee on Cancer; HR: hazard ratio; CI: confidence interval; Ref: reference
